# Mid-term Outcomes in Primary Roux-en-Y Gastric Bypass Procedures with Short or Long Biliopancreatic Limb

**DOI:** 10.1007/s11695-025-08263-z

**Published:** 2025-09-22

**Authors:** Julian Süsstrunk, Svenja Erne, Alexander Wilhelm, Thomas Köstler, Diana Mattiello, Urs Zingg

**Affiliations:** 1 Limmattal Hospital, Zurich-Schlieren, Switzerland; 2 University Digestive Health Care Center, St. Clara Hospital and University Hospital Basel, Basel, Switzerland

**Keywords:** Bariatric surgery, Gastric bypass, Limb length, Type 2 diabetes

## Abstract

**Background:**

This study reports mid-term results on weight loss and T2D remission in patients undergoing Roux-en-Y gastric bypass (RYGB) with short versus long biliopancreatic limb (BPL).

**Methods:**

All patients with obesity undergoing RYGB procedures with long BPL (150 cm) versus short BPL (60 cm) between 2016 and 2021 at a tertiary reference center for bariatric surgery were compared using propensity score matching to assess for T2D remission, HbA1c evolution, weight loss and nutritional deficiencies.

**Results:**

A total of 165 patients were included, 69 patients (71% female, mean age 43.9 ± 14 years, mean baseline BMI 42.4 ± 4.3 kg/m^2^, mean HbA1c 6.75 ± 1.9%) underwent long-BPL RYGB and 96 patients (76% female, mean age 43.1 ± 12 years, mean baseline BMI 41.7 ± 3.5 kg/m^2^, mean HbA1c 5.97 ± 1.1%) had a short-BPL. In long-BPL RYGB weight loss was more accelerated after 1 year, but did not differ after 2 years compared to short-BPL RYGB (mean %TWL of 33.9 ± 9.4% versus 31.6 ± 7.7%; *p* = 0.09 at 1 year and mean %TWL of 33.8 ± 9.6% and 32 ± 8.8%; *p* = 0.24 at 2 years). Complete remission of T2D occurred in 53.3% after long-BPL RYGB and 61.3% after short-BPL RYGB (*p* = 0.53). Long-BPL RYGB resulted in higher reduction of HbA1c after 2 years (1.5% vs. 0.72%, *p* =  < 0.001). Besides a higher zinc deficiency in the long-BPL group (*p* = 0.009), no significant differences in nutritional deficiencies or malnutrition were observed between the two groups.

**Conclusion:**

Implementation of a long-BPL RYGB is safe and shows an accelerated weight loss and improved HbA1c reduction with low overall morbidity after 2 years.

## Introduction

The prevalence of obesity has dramatically increased over the last decades, affecting individuals across all age groups [1]. Sleeve gastrectomy (SG) is the most frequently performed bariatric procedure, followed by Roux-en-Y gastric bypass (RYGB) and one anastomosis gastric bypass (OAGB) [2]. In the context of obesity, type 2 diabetes mellitus (T2D) is a relevant associated medical condition due to its associated long-term complications. Its prevalence is increasing in correlation with the prevalence of obesity [3]. A landmark study comparing metabolic bariatric surgery (MBS) with conventional medical treatment demonstrated that RYGB and biliopancreatic diversion (BPD) were more effective in achieving long-term control of T2D compared to best medical treatment [4]. When looking at the most commonly performed procedure SG, a randomized trial by Svanevik et al. reported inferior outcomes regarding weight loss and remission of T2D three years after SG compared to RYGB [5]. Moreover, SG seems not only inferior in terms of weight loss and HbA1c reduction, but also in the reduction of associated cardio-vascular conditions and remission of dyslipidemia in patients with T2D [6, 7].

Although RYGB is the preferred procedure in patients with obesity and T2D, the limb lengths in RYGB regarding optimal control of T2D and weight loss remain controversial [8]. Increased length of the biliopancreatic limb (BPL) in RYGB improved the anti-diabetic effect and resulted in enhanced weight loss. The rationale for lengthening the BPL instead of the Roux limb (RL) is based on findings, that bypassing more proximal small bowel leads to enhanced secretion of incretin hormones, such as glucagon-like-peptide 1 (GLP-1) and peptide tyrosine tyrosine (PYY) [9]. These changes, in conjunction with increased circulating bile acid concentrations, are considered key mechanisms contributing to improved glycemic control and weight loss after RYGB [10–12]. However, most previous studies reported only short-term outcomes and had variable definitions for the length of the BPL [13–16]. The lengthening of the BPL is desirable concerning the weight loss and T2D resolution. However, it may come at the cost of potential nutritional side effects.

The optimal limb lengths for patients with T2D have not been determined yet and the investigation on BPL length for optimal weight loss and improvement of obesity-related conditions is of importance. Thus, the aim of this study was to report on mid-term weight loss outcomes and T2D evolution of patients undergoing RYGB with a standard versus a long BPL.

## Methods

### Design and Patients

Data of all patients undergoing a bariatric procedure at the XXX (blinded), is entered into a prospective database. For this retrospective, non-randomized, matched case cohort study, all patients undergoing laparoscopic RYGB between January 2016 and December 2021 were analyzed. Patients with previous bariatric procedures such as adjustable gastric banding or SG were excluded. To assess the effect of variable BPL lengths, patients were divided into two groups: Short-BPL RYGB consisted of a BPL of 60 cm and a Roux limb of 150–200 cm. Long-BPL RYGB consisted of a BPL of 150 cm and a RL of 100–150 cm.

### Preoperative Assessment, Surgical Technique, and Perioperative Care

All patients were assessed preoperatively by a multidisciplinary team according to the guidelines of the Swiss Multidisciplinary Obesity Society (SMOB). Preoperative evaluation included upper endoscopy with biopsy, abdominal ultrasound and assessments by dieticians, endocrinologists, cardiologists, psychologists and pulmonologists, respectively.

Selection of the procedure was dependent on the outcome of the bariatric multidisciplinary team discussion as well as the expectations and wishes of the patients. All patients with T2D, regardless of preoperative BMI, were evaluated for a long-BPL RYGB.

Both short and long-BPL RYGB were performed using a six-port technique. A small gastric pouch (approx. 40–50 cc) was created and an antecolic circular or linear stapled gastro-enterostomy was formed. In short-BPL RYGB the RL measured 150–200 cm and in long-BPL RYGB 100–150 cm. Intraoperative measurement of the limb lengths was performed using the length of the gaspers’ end (approx. 30 mm) as a reference for laparoscopic measuring. With a side-to-side, linear stapled entero-enterostomy, a BPL of 60 cm in short-BPL RYGB and 150 cm in long-BPL RYGB was fashioned. Total bowel length or common channel length was not measured routinely. Mesenteric defects were closed with non-resorbable sutures in all procedures.

Independent of the procedure choice, the patients received equal postoperative multivitamin, calcium and vitamin D supplementation in the beginning, adjusting the dose according to blood tests during the follow-up visits. These regular postoperative follow-up visits were scheduled after 6 weeks, 6 months, 12 months and yearly thereafter.

### Outcome Measures

The primary outcome was weight loss expressed as percentage total weight loss (%TWL) calculated as follows: [(initial weight) − (postoperative weight)]/[(initial weight)].

Secondary outcomes were weight loss expressed as reduction of BMI, T2D remission, defined as HbA1c value of 5.6% or less, without the use of any antidiabetic medication, a stricter definition as the one proposed by the American Diabetes Association [17]. For the current study, we differentiated between prediabetes with a HbA1c value ranging from 5.7% up to 6.4% and T2D, defined by a HbA1c value of 6.5% or higher. Further secondary outcomes were percentage reduction of HbA1 value (*ΔHbA1c; (initial HbA1c − postoperative HbA1c) in %*), malnutrition (*serum albumin* < *35 g/L*), vitamin and substrate deficiencies. Additionally, presence of obesity-related conditions such as arterial hypertension (AT) (*systolic blood pressure* > *140 mmHg with/without use of antihypertensive medication*), sleep apnea confirmed by a pulmonologists examination, coronary heart disease confirmed by a cardiologist’s examination, peripheral artery disease (PAD) confirmed by a vascular specialists examination and hyperlipidemia (*Cholesterol* > *6.2 mmol/L, triglycerides* > *1.2 mmol/L*) were assessed. Analysis of vitamin and substrate deficiencies included vitamin A (< *0.3 mg/L*), Vitamin D (< *31* $\mu$ *g/L*), Vitamin B1 (< *33.1* $\mu$ *g/L*), B6 (< *12.6* $\mu$ *g/L*) and B12 (< *145 pmol/L*), holotranscobalamin (< *37.5 pmol/L*), zinc (< *9* $\mu$ *mol/L*), albumin corrected calcium (< *2.15 mmol/L*), hypoproteinemia (*serum prealbumin* < *200 mg/L*), ferritin (< *13* $\mu$ *g/L*) and folic acid in erythrocytes (< *523* $\mu$ *g/L*). Moreover, use of vitamin and/or substrate supplementation preoperatively and use of supplementation exceeding the routinely administered supplementation dosage postoperatively also counted as a deficiency.

#### Statistical Analysis

Data are presented either as mean ± standard deviation or, in the case of proportions, as numbers (%). Differences between groups were compared using the two sample *t* test for continuous data and Pearson’s Chi-square test for categorical data. No formal corrections for multiple comparisons were applied, consistent with recommendations for exploratory analyses. Normality of continuous variables was visually assessed using histograms and Q–Q plots.

To account for differences between groups, patients underwent propensity score matching using the following matching criteria: sex, age, body mass index (BMI) and presence of Type 2 Diabetes. Propensity scores were estimated via logistic regression, and 1:2 nearest-neighbor matching without replacement was applied using a caliper width of 0.2 standard deviations of the logit of the propensity score. Since this study is a retrospective analysis of a prospectively maintained clinical database, a complete-case-analysis approach was used ensuring that only complete datasets were included in the analysis.

A *p* value of 0.05 was considered significant. A formal power calculation was not performed, and the sample size was determined by the number of patients meeting the inclusion criteria during the study period. All statistical analyses were performed using R Programming language (version 4.4.1, R Core Team 2024).

## Results

### Patient Characteristics

**Fig. 1:**
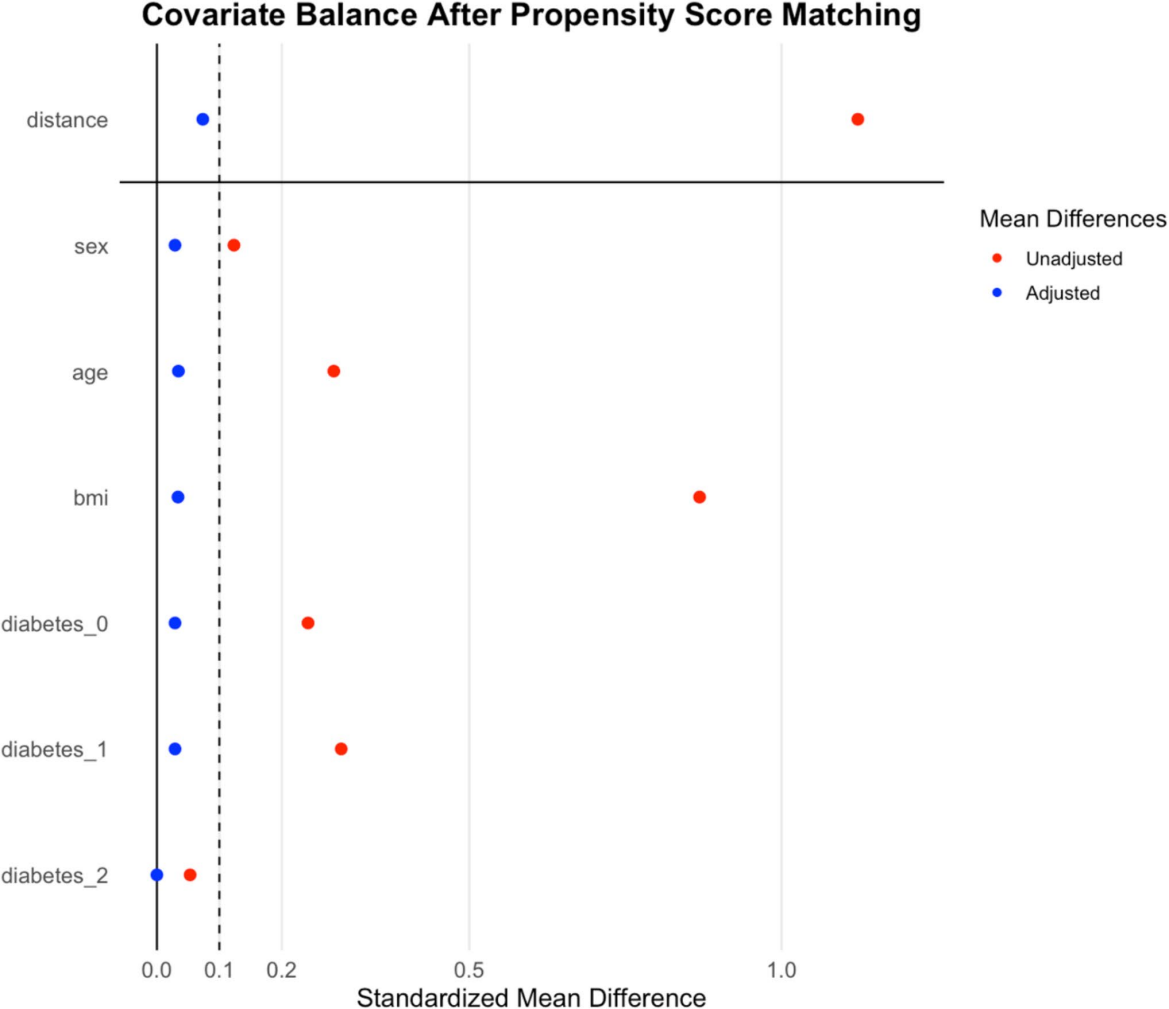
Covariate balance after propensity score matching

**Fig. 2:**
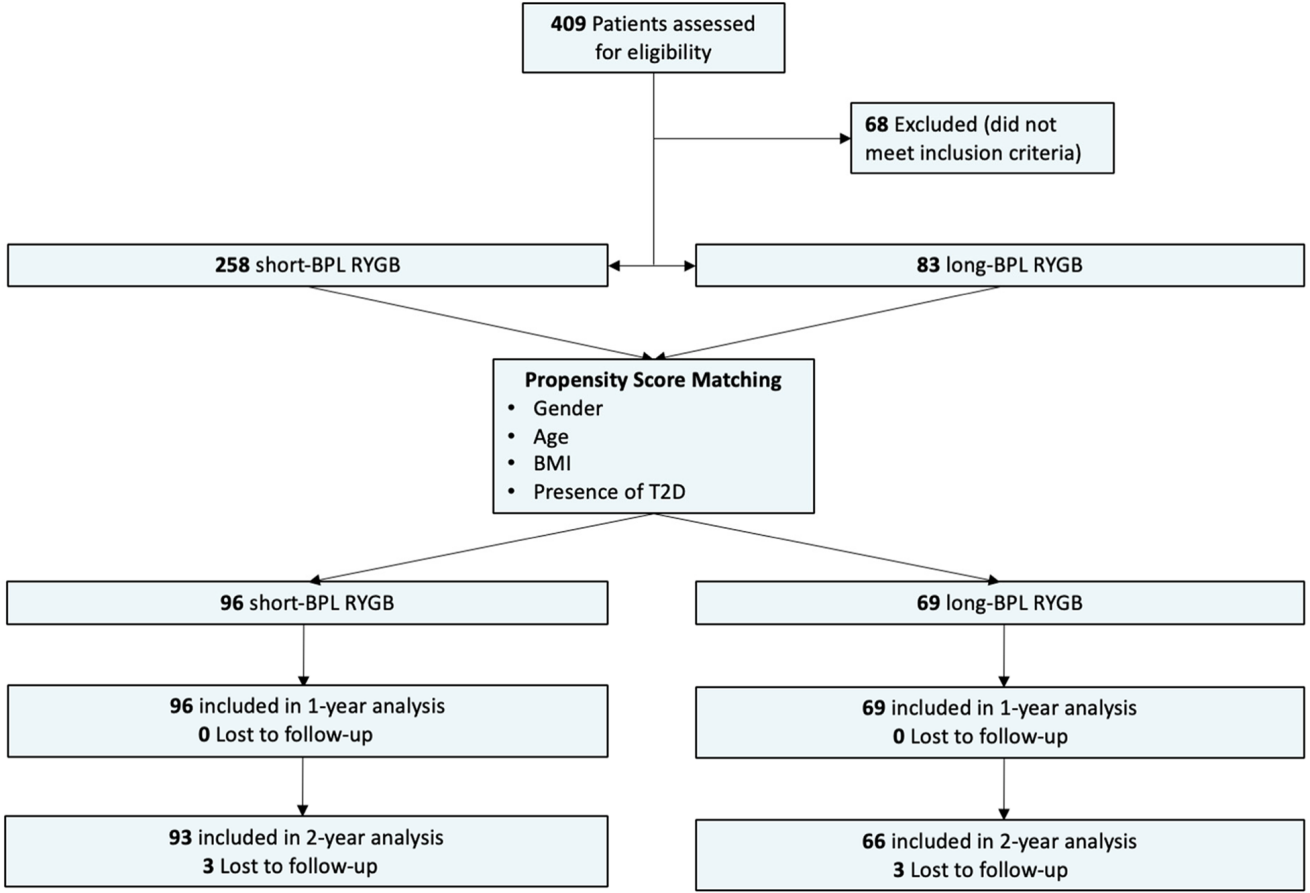
Flow chart describing patient selection

To maximize comparability, a 1:2 matching ratio was used, resulting in 69 long-BPL RYGB patients (71% female, mean age 43.9 ± 14 years, mean baseline BMI 42.4 ± 4.3 kg/m^2^) being matched to 96 short-BPL RYGB patients (76% female, mean age 43.2 ± 12 years, mean baseline BMI 41.7 ± 3.5 kg/m^2^). The remaining unmatched patients were excluded from the matched analysis. Balance diagnostics demonstrated standardized mean differences <0.1 for all matched covariates, indicating excellent covariate balance (Figure 1). The propensity score itself (“distance”) showed a post-matching standardized mean difference of 0.074, further supporting the adequacy of the matching procedure. Short-BPL RYGB consisted of a BPL of 60 cm and a Roux limb (RL) of 150–200 cm. Long-BPL RYGB consisted of a BPL of 150 cm and a RL of 100–150 cm. Therefore, total bypassed limb length was comparable in both groups (210–260 cm versus 250–300 cm respectively). There were no significant differences between the groups concerning gender, age or preoperative BMI. Figure 2 displays the cohort after matching and provides information on follow-up data. All 165 operations were performed laparoscopically; no conversion to open surgery occurred. In the short-BPL RYGB group, 34% had a T2D and 16% had a prediabetes, compared to 45% and 14% in the long-BPL group (*p* = 0.38). Forty-three percent of patients used antidiabetic drugs in the long-BPL group compared to 27% in the short-BPL group (*p* = <0.001). Baseline HbA1c levels were 6.75 ± 1.9 and 5.97 ± 1.1 in the long- and short-BPL group, respectively (*p* = 0.001). Detailed information on comorbidities and baseline laboratory results are shown in Table 1.

**Table 1:** Baseline characteristics

	Short-BPL RYGB (*n* = 96)	Long-BPL RYGB (*n* = 69)	*p* value
Patient sex			0.47
Female	73 (76%)	49 (71%)	
Male	23 (24%)	20 (29%)	
Age at operation, year, mean (SD)	43.15 (12.14)	43.88 (13.71)	0.72
Baseline weight, kg, mean (SD)	114.83 (15.12)	119.249 (17.29)	0.083
Baseline BMI, kg/m^2^, mean (SD)	41.68 (3.50)	42.43 (4.33)	0.22
Excess weight, kg, mean (SD)	45.84 (10.46)	48.93 (12.74)	0.090
Baseline albumin deficiency	0 (0%)	0 (0%)	
Baseline ferritin deficiency	2 (2%)	2 (3%)	0.74
Baseline hypo- vs. hyperthyroidism			0.35
Hypothyroidism	1 (1%)	0 (0%)	
Hyperthyroidism	0 (0%)	1 (1%)	
Baseline folic acid in erythrocytes deficiency	3 (3%)	4 (6%)	0.38
Baseline calcium deficiency	8 (8%)	4 (6%)	0.54
Baseline zinc deficiency	3 (3%)	2 (3%)	0.92
Baseline vitamin B12 deficiency	0 (0%)	0 (0%)	
Baseline holotranscobalamin deficiency	6 (7%)	6 (9%)	0.54
Baseline vitamin A deficiency	1 (1%)	1 (2%)	0.80
Baseline vitamin D3 deficiency	89 (95%)	67 (97%)	0.45
Baseline vitamin B1 deficiency	0 (0%)	0 (0%)	
Baseline vitamin B6 deficiency	49 (55%)	34 (54%)	0.89
Baseline Diabetes			0.38
Prediabetes	33 (34%)	31 (45%)	
Diabetes	15 (16%)	10 (14%)	
Baseline HbA1c, mean (SD)	5.97 (1.09) %	6.75 (1.89) %	0.001
Baseline diabetes drugs			< 0.001
OAD	23 (24%)	12 (17%)	
OAD + insulin	1 (1%)	5 (7%)	
Insulin	2 (2%)	13 (19%)	
Baseline hypertension	41 (43%)	40 (59%)	0.042
Baseline hypertension drugs	35 (36%)	32 (46%)	0.20
Baseline coronary heart disease	6 (6%)	5 (7%)	0.80
Baseline PAD	1 (1%)	1 (1%)	0.81
Baseline hyperlipidemia	34 (35%)	31 (45%)	0.22
Baseline hyperlipidemia drugs	16 (17%)	14 (20%)	0.55
Baseline OSAS	44 (46%)	40 (58%)	0.12
Baseline CPAP therapy	14 (15%)	16 (23%)	0.16

() = % if not otherwise stated

*RYGB* Roux-en-Y gastric bypass, *BMI* body mass index, *OAD* oral antidiabetics, *PAD* peripheral artery disease, *OSAS* obstructive sleep apnea syndrome, *CPAP* continuous positive airway pressure. *SD* standard deviation

### BMI Changes and Total Weight Loss

**Fig. 3:**
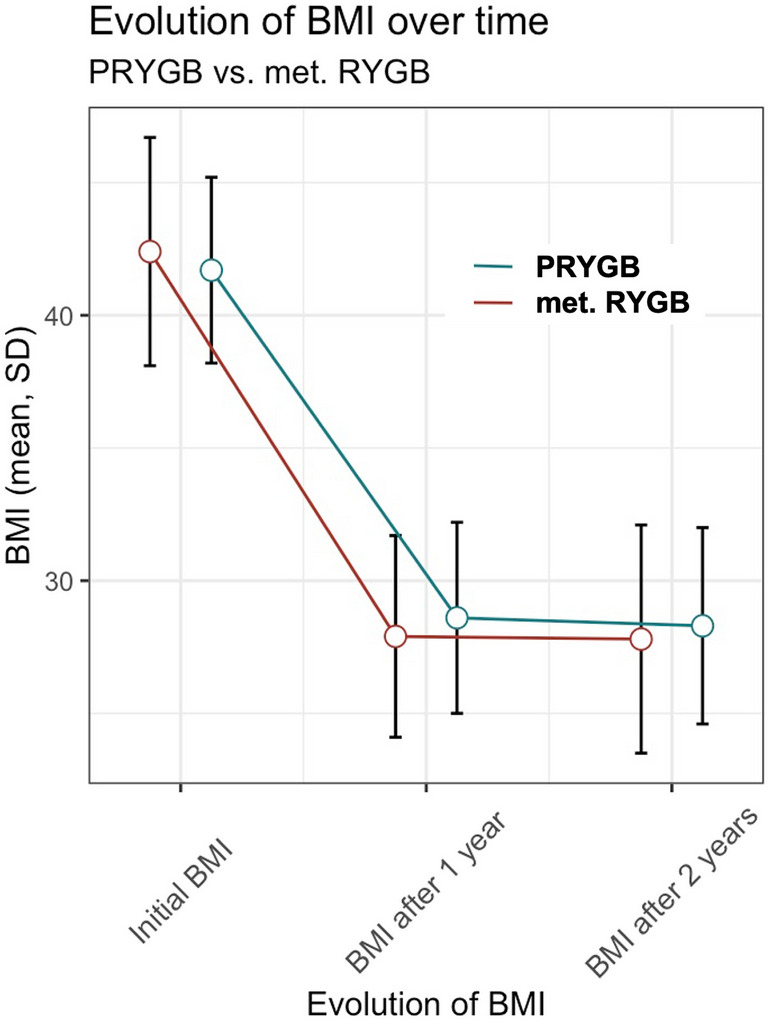
Evolution of BMI over time;* p value 1 year postoperative* = *0.028, p value 2 years postoperative* = *0.139; sample size 1 year postoperative: short-BPL n* = *96, long-BPL n* = *69; sample size 2 years postoperative: short-BPL n* = *93, long-BPL n* = *66*

Data on postoperative weight loss are shown in Table 2. After 1 year, %TWL was 31.57 ± 7.66% in the short-BPL RYGB group and 33.86 ± 9.43% in the long-BPL RYGB group (*p* = 0.088), and after 2 years 32.04 ± 8.83% in the short-BPL RYGB group and 33.78 ± 9.75% in the long-BPL RYGB group (*p* = 0.242). At 1-year follow-up, the mean weight loss was significantly higher in the long-BPL group with 44.8 ± 33.1 kg compared to 36.4 ± 10.5 kg in the short-BPL RYGB group (*p* = 0.021). After 2 years, this difference was not significant anymore (36.9 ± 11.5 kg in the short-BPL RYGB group and 40.3 ± 13.3 kg in the long-BPL RYGB group, *p* = 0.092). Figure 3 illustrates the evolution of the BMI over time in the two groups, demonstrating an accelerated BMI reduction in the long-BPL RYGB group.

**Table 2:** Weight loss parameters over the study period

	Short-BPL RYGB *(n* = *96)*	Long-BPL RYGB *(n* = *69)*	*p* value
Weight loss/BMI changes at 1 year			
BMI (kg/m^2^)	28.57 (3.58)	27.86 (3.81)	0.22
Weight (kg)	78.48 (12.9)	78.83 (15.99)	0.87
Weight loss (kg)	36.42 (10.53)	44.85 (33.14)	0.021
BMI reduction (kg/m^2^)	13.12 (3.85)	14.58 (4.58)	0.028
TWL (%)	31.57 (7.66)	33.86 (9.43)	0.088
Weight loss/BMI changes at 2 years			
BMI (kg/m^2^)	28.3 (3.7)	27.82 (4.3)	0.46
Weight (kg)	77.96 (13.74)	78.86 (16.88)	0.71
Weight loss (kg)	36.9 (11.51)	40.25 (13.32)	0.092
BMI reduction (kg/m^2^)	13.42 (4.06)	14.49 (4.95)	0.139
TWL (%)	32.04 (8.83)	33.78 (9.75)	0.242

Values are shown as median (standard deviation)

*BMI* body mass index, *%TWL* total weight loss

### Remission of Type 2 Diabetes and Evolution of Hba1c Levels

Data on postoperative T2D are shown in Table 3. At 1-year follow-up, T2D was present in 7% and in 17% in the short-BPL and long-BPL groups, respectively. Prediabetes occurred in 8% of patients in the short-BPL RYGB group compared to 3% in the long-BPL RYGB group. After 2 years follow up, 5% of patients still had a T2D and 9% a prediabetes after short-BPL RYGB compared to 14% and 9% in the long-BPL RYGB group. This corresponds to a remission rate of 61.3% in the short-BPL RYGB group and 53.3% in the long-BPL RYGB group, respectively (*p* = 0.53). Discontinuation of antidiabetic drugs at 1 year follow up was observed in 88.5% in the short-BPL RYGB and in 70% in the long-BPL RYGB group (*p* = 0.092). At the 2-year follow-up, the discontinuation rates were 91.7% and 72.4 %, respectively (*p* = 0.075).

**Table 3:** Diabetes parameters over the study period

	Short-BPL RYGB *(n* = *96)*	Long-BPL RYGB *(n* = *69)*	*p* value
Diabetes changes at 1 year			
Diabetes/prediabetes			0.06
Diabetes (%)	7 (7%)	12 (17%)	
Prediabetes (%)	8 (8%)	2 (3%)	
HbA1c (%)	5.16 (0.71)	5.25 (0.7)	0.43
ΔHbA1c (%)	0.79 (1.05)	1.5 (1.69)	0.001
Diabetes drugs			0.086
OAD (%)	2 (2%)	7 (10%)	
Insulin (%)	1 (1%)	2 (3%)	
Diabetes changes at 2 years			
Diabetes/prediabetes			0.19
Diabetes (%)	5 (5%)	9 (14%)	
Prediabetes (%)	8 (9%)	6 (9%)	
HbA1c (%)	5.22 (0.5)	5.28 (0.82)	0.60
ΔHbA1c (%)	0.72 (0.92)	1.51 (1.73)	< 0.001
Diabetes drugs			0.031
OAD (%)	2 (2%)	6 (9%)	
Insulin (%)	0 (0%)	2 (3%)	

Values are shown as mean (standard deviation)

*OAD* oral antidiabetic drugs

Considering that the preoperative HbA1c levels were significantly higher in the long-BPL RYGB group compared to the short-BPL RYGB group, ΔHbA1c was additionally analyzed postoperatively. At 1-year follow-up, ΔHbA1c was significantly lower in the short-BPL RYGB group (0.79 ± 1%) compared to the long-BPL RYGB group (1.5 ± 1.7%, *p* = 0.001). This significant difference persisted at 2-year follow-up with a short-BPL RYGB ΔHbA1c of 0.71 ± 0.9% and long-BPL RYGB ΔHbA1c of 1.5 ± 1.7% (*p* < 0.001). However, mean HbA1c levels at 1-year follow up were 5.16 ± 0.71% in the short-BPL group and 5.25 ± 0.7% in the long-BPL group (*p* = 0.43). HbA1c levels at the 2-year follow up were 5.22 ± 0.5% and 5.28 ± 0.82% (*p* = 0.60) respectively. This indicates no significant difference in glycemic control despite a greater reduction in the long-BPL group.

### Resolution of Obesity-Related Medical Conditions and Nutritional Deficiencies

No difference in the resolution of the various obesity-related medical conditions was detected between the groups. Resolution of arterial hypertension (AT) occurred in 71% in the short-BPL RYGB group and in 68.4% in the long-BPL RYGB group (*p* = 0.8). Resolution of hyperlipidemia was observed in 81.3% in the short-BPL RYGB group and in 80% in the long-BPL RYGB group (*p* = 0.9). Obstructive sleep apnea syndrome (OSAS) was eliminated in 92.9% of the short-BPL RYGB patients and in 87.2% of the long-BPL RYGB patients (*p* = 0.392).

Malnutrition occurred in two patients at 2-year follow-up, both with a long-BPL RYGB. With an albumin of 31 g/L and 33 g/L respectively, neither patient required inpatient treatment (*p* = 0.091).

An overview of the comparison of vitamin and substrate deficiencies at the 2-year follow-up is demonstrated in Table 4. There was a significant difference in the incidence of zinc deficiency with 22% of patients following short-BPL RYGB and in 41% of patients after long-BPL RYGB, respectively (*p* = 0.009). There was no significant difference among all other vitamin and substrate deficiencies between groups.

**Table 4:** Nutritional parameters at 2-year follow-up

	Short-BPL RYGB *(n* = *96)*	Long-BPL RYGB *(n* = *69)*	*p* value
Albumin deficiency	0 (0%)	2 (3%)	0.091
Ferritin deficiency	34 (37%)	25 (38%)	0.87
Hypo- vs. hyperthyroidism			0.23
Hypothyroidism	4 (4%)	6 (9%)	
Hyperthyroidism	0 (0%)	1 (2%)	
Deficiency of folic acid in erythrocytes	5 (5%)	3 (5%)	0.81
Calcium deficiency	13 (14%)	13 (20%)	0.34
Zinc deficiency	20 (22%)	26 (41%)	0.009
Holotranscobalamin deficiency	27 (30%)	21 (32%)	0.72
Vitamin A deficiency	4 (5%)	6 (10%)	0.22
Vitamin D3 deficiency	64 (69%)	53 (80%)	0.11
Vitamin B1 deficiency	2 (2%)	1 (2%)	0.79
Vitamin B6 deficiency	16 (17%)	11 (17%)	0.99

() = %

### Length of Hospital Stay and Complications

No death related to bariatric surgery occurred during the entire follow-up. One patient died 2 years and 9 months postoperative due to hepatocellular carcinoma unrelated to surgery. In most cases hospital stay ranged from 2 to 4 days. Longest hospital stay was 44 days due to complications including bleeding from the entero-enterostomy, backlog of the BPL, aspiration pneumonia, and pulmonary embolism necessitating 18 days of ICU care. An overview of the postoperative complications classified by Clavien-Dindo of at least grade IIIa is shown in Table 5. Fifteen patients (15.6%) had a grade 3a or higher complication after short-BPL RYGB and 19 patients (27.5%) after long-BPL RYGB (*p* = 0.095).

**Table 5:** Overview of postoperative complications classified by Clavien-Dindo

Complication	Clavien-Dindo grade	Notes	Patients (*n*)	Short-BPL (*n*)	Long-BPL (*n*)
Time frame < 30 days					
Abdominal pain with preoperative diagnosis of cholecystolithiasis and intraoperative diagnosis of an adhesive band	IIIb	Laparoscopic adhesiolysis and laparoscopic cholecystectomy	1	1	
Douglas abscess	IIIb	Laparoscopic abscess drainage	1	1	
Leak of the entero-enterostomy	IIIb	Laparoscopic revision of the entero-enterostomy	1		1
Wound abscess	IIIa	Treated with antibiotics and CT-guided drainage	1	1	
Bleeding from the stapler line on the residual stomach	IIIb	Laparoscopic hemostasis	1		1
Entero-enterostomy bleeding with backlog in the BPL leading to perforation of the residual stomach	IVa	Laparoscopic hemigastrectomy, enterostomy, revision of the entero-enterostomy and abdominal lavage	1	1	
Bleeding of the entero-enterostomy with backlog of the BPL followed by aspiration pneumonia and pulmonary embolism necessitating ICU-Care	IVb	Laparoscopic revision of the entero-enterostomy and intraoperative Gastroscopy	1		1
Time frame > 30 days					
Chronic/recurrent anastomotic ulcer	IIIb	Laparoscopic revision of the gastro-enterostomy	6	3	3
Ulcer bleeding	IIIb	Laparoscopic formation of an esophago-jejunostomy	1		1
Bleeding in the residual stomach caused by recurrent ulcers in the antrum	IIIb	Laparoscopic assisted gastroscopy and gastrectomy of the residual stomach	1	1	
Stenosis of the gastro-enterostomy	IIIa	Managed endoscopically by bougienage	1		1
Gastro-gastric fistula	IIIb	Laparoscopic revision of the gastro-enterostomy with partial resection of the gastric pouch and resection of the gastric fundus	1		1
Jejunal ulcer distal to the gastro-enterostomy	IIIb	Laparoscopic revision of the gastro-enterostomy	1	1	
Symptomatic cholecystolithiasis	IIIb	Laparoscopic cholecystectomy	2	2	
Internal hernia of Petersen’s space with/without incarceration	IIIb	Reposition of the bowel and closure of the hernia with/without segmental resection of the small bowel	6	1	5
Diffuse abdominal pain due to (partially) open intermesenteric defects	IIIb	Closure of the intermesenteric defects	3	3	
Diffuse abdominal pain related to cholecystolithiasis and adhesive bands	IIIb	Laparoscopic adhesiolysis and cholecystectomy	1		1
Diffuse abdominal pain related to kinking and dilation of the entero-enterostomy	IIIb	Laparoscopic revision of the entero-enterostomy	1		1
Perforation of the entero-enterostomy related to a bezoar	IIIb	Open revision of the entero-enterostomy	1		1
Incisional hernia	IIIb	Open hernia reposition and sublay mesh repair with partial component separation	1		1
Adhesive small bowel obstruction	IIIb	Laparoscopic adhesiolysis	1		1
Total (*n*)			34 (20.6)	15 (15.6)	19 (27.5)

() = %; *p* = 0.095

## Discussion

Our study revealed no significant difference in weight loss outcomes between short-BPL RYGB and long-BPL RYGB at 2 years with a %TWL of 32.04 ± 8.83% and 33.78 ± 9.75%, respectively. However, in the long-BPL group, the weight loss was more accelerated in the first year. Regarding T2D remission, we observed no significant difference at 2 years in both groups despite more patients with insulin-dependent T2D and higher HbA1c levels preoperatively in the long-BPL group. Nevertheless, the long-BPL RYGB was associated with an improved metabolic response, reflected in a greater decrease in ΔHbA1c. Hence, a longer BPL facilitates a greater drop in blood sugar levels in patients with poorer initial glycemic control. However, no significant difference was observed in HbA1c levels at 1 and 2 years.

These findings suggest that the BPL may be elongated even more to increase the weight loss and improve the resolution of obesity-related medical conditions especially T2D. However, before changing limb lengths arbitrarily it is crucial to understand the altered gastrointestinal physiology after bariatric surgery. The RYGB is widely known to induce weight loss and remission of obesity-related medical conditions through a combination of gastric restriction and intestinal bypass that alters nutrient absorption and gut signaling. More recent literature evaluates different mechanisms in gastrointestinal physiology leading to these effects. Among other factors, elevated bile acid levels and altered release of gut hormones are identified as contributors to an increased effectiveness of bariatric surgery [18, 19]. The gut hormone glucagon-like-peptide 1 (GLP-1), which is released postprandially by L-cells in the ileum, is assumed to play a key role in improved postoperative glucose tolerance via the incretin effect [10]. Furthermore, by different pathways, GLP-1 superiorly acts as a satiety hormone, hence leading to weight loss [20]. The same effects are seen in GLP-1 receptor agonists administration in treatment of obesity and T2D [21, 22]. GLP-1 secretion and also peptide tyrosine tyrosine (PYY) secretion, another incretin, are elevated in any situation of accelerated gastric emptying and rapid transport of undigested nutrients to a more distal part of the small intestine, such as it is the case in RYGB patients [9]. Further data from a rat model highlighted the essential contribution of PYY-release on GLP-1 secretion [23]. In regard to serum bile acids, their levels are also elevated following RYGB [24]. It is hypothesized that elevated bile acid levels lead to an improved glycemic control. However, the exact underlying mechanisms are poorly understood [11]. By elongating the BPL, a rise in GLP-1 levels could be demonstrated in humans and rat models [25, 26]. Furthermore, concentrations of serum bile acids also seem to be increased with a longer biliopancreatic limb [11].

Taking our results in light of the current aforementioned literature, the long BPL achieves faster weight loss, even though the total bypassed bowel length (BPL + Roux limb) stayed more or less the same in both groups at approximately 250–260 cm. Furthermore, a better outcome with regard to the reduction of the HbA1c level could be observed. However, no significant difference in the T2D remission rate was observed between the two groups. To gain a higher percentage of T2D remission, a further lengthening of the BPL needs to be considered. In a study conducted by Zerrweck et al. the BPL was lengthened up to 200 cm. In the short-term follow up of 12 months, weight loss (% EWL) and reduction of HbA1c levels were increased in comparison to a short BPL of 50 cm [14]. However, similar to our results, the clinical remission rate of T2D was comparable between groups, and the effect was only seen in the significantly higher decrease of HbA1c levels. Unfortunately, the study lacked long-term results. Another analysis of a 200 cm BPL demonstrated improved metabolic outcomes compared to BPL with a length of 84 ± 2 cm, reflected by higher T2D remission and lower antidiabetic drug requirement [27].

A potential factor influencing T2D remission rate is the duration of the disease at the time of bariatric surgery, and the time of its onset. In a short-term follow-up, a better glycemic control was achieved with gastric bypass procedures in patients with an early-onset T2D (<40 years of age) compared to patients with a late-onset T2D (≥40 years of age) [28]. Factors predicting a poorer postoperative T2D outcome are age over 40 to 50 years, higher HbA1-levels and insulin-use. Also, a higher BMI indicates a higher probability of T2D remission [29]. Moreover, longer T2D duration is a poor predictor for remission. However, it remains unclear at what level the threshold for a better postoperative outcome should be set at [30, 31]. Unfortunately, our data set did not allow to analyze the time frame of T2D onset and duration.

According to the American Society of Metabolic and Bariatric Surgery (ASMBS) and the International Federation for the Surgery of Obesity and Metabolic Disorders (IFSO), MBS is generally recommended in patients with a BMI ≥ 35kg/m^2^, whereas in patients with T2D it’s already recommended with a BMI of ≥30kg/m^2^ [32]. Based on our results and the actual literature, the conclusion that a longer BPL induces accelerated weight loss and improved T2D control without relevantly triggering malnutrition is sound. From a clinical perspective, the accelerated initial weight loss observed after long-BPL RYGB may be relevant in selected patient groups. Patients with poorly controlled T2D, a high insulin dose requirement, advanced cardiovascular risk or severe obesity could benefit from more rapid metabolic improvement. Furthermore, Silveira et al. demonstrated, that TWL of <10% at 3 months is associated with suboptimal weight loss at 1 year [33]. Despite the absence of a significant difference in weight loss in our study population at the 2-year follow-up, these early advantages may justify the use of a longer BPL in carefully chosen patients. However, long-term RCTs are required to confirm whether such patient stratification should be systematically implemented in daily practice.

The adverse effects like diarrhea did not differ between the two BPL groups. Malnutrition was rare. Zinc deficiency was the only nutritional deficiency that was significantly higher in the long-BPL RYGB group compared to the short-BPL group. Zinc absorption occurs in the proximal small bowel by zinc transporters, which are regulated by multiple factors [34]. Lengthening of the BPL leads to a longer bypass of the proximal small bowel, which explains this higher rate of zinc deficiency in long-BPL RYGB. A study conducted by Thereaux et al. demonstrated increased late adverse events such as higher nutritional disorders in RYGB and SG compared to a control group, however in favor of a decreased 7-year overall mortality [35]. Data on nutritional deficiencies in patients with different lengths of the BPL are scarce and partially contradicting. Zerrweck et al. found no difference in hemoglobin or albumin levels as indirect parameters for malnutrition [14]. In contrast, Nergaard et al. reported significantly higher requirements for supplementation of vitamin D, iron and calcium citrate in a long BPL of 200cm compared to a short BPL of 60 cm with a follow-up up to 7 years [36]. Given that nutritional deficiencies can be monitored and supplemented, bariatric surgery is associated with better long-term outcomes than medical treatment for obesity. A longer BPL in RYGB should be considered as it may further improve outcomes concerning weight loss and resolution of obesity-related conditions. However, it should be recognized that the follow-up and nutritional monitoring of RYGB patients, especially if a longer BPL is used, must be performed over a long-time if not life-long.

Limitations of this study consist of the retrospective nature and the mid-term follow-up of 2 years. However, all data were collected prospectively and, due to the propensity score matching, the two groups were comparable at baseline. The sample size of 165 patients may have been insufficient to detect smaller but clinically relevant differences, such as in the T2D remission rate. Since no a priori power calculation was performed and sample size was determined by patient availability, the risk of type II errors must be considered. Therefore, non-significant findings should not be interpreted as equivalence. The values and findings remained stable between the 1 and 2-year follow-up. Unfortunately, onset and duration of T2D were not assessed in the study population. The 2-year follow-up of the present study is longer than in most other reports discussing different BPL lengths. Furthermore, the rate of patients lost to follow-up was exceptionally low. However, studies with long-term follow-up over several years are of interest because differences in weight loss, diabetes remission and nutritional deficiencies are assumed to occur only over a longer time period. The systematic review and meta-analysis by Rizvi et al. emphasize this point [37]. As this is not a randomized study, a certain selection bias could affect the results. Patients with a higher HbA1c level and insulin-dependency were more likely selected for a long-BPL RYGB. Although propensity score matching accounted for the presence of T2D and prediabetes, it did not include HbA1c levels or insulin dependency. These remaining differences may have influenced glycemic outcomes. Therefore, one needs to interpret postoperative results on HbA1c changes with caution. Furthermore, we did not specifically analyze outcomes within subgroups due to the matched cohort design and limited sample size. Other limitations include that the total bowel length was not measured, so anatomical differences were not accounted for. Additionally, there was no difference in diarrhea between the two groups. However, no formal Quality of Life (QoL) or gastrointestinal symptom analysis was performed. Finally, as this was a single-center study, the results may not be generalizable to all populations or surgical settings. Nevertheless, our findings align with those of other published studies, suggesting broader applicability. However, bariatric surgery warrants tailoring to the individual patient’s characteristics and the present cohort represents the clinical daily life in bariatric surgery. Still, further studies, in particular those of a prospective randomized controlled nature, are of importance such as the announced SLIM trial, of which the results are expected to be published in the future [38].

In conclusion, implementation of a long-BPL RYGB with a BPL of 150 cm and an RL of 100–150 cm is safe. It provides accelerated weight loss and improved metabolic response as reflected by HbA1c reduction with low overall morbidity and manageable nutritional deficiencies.

## Data Availability

Research data supporting this publication is available upon reasonable request.

## References

[1] Phelps NH, Singleton RK, Zhou B, et al. Worldwide trends in underweight and obesity from 1990 to 2022: a pooled analysis of 3663 population-representative studies with 222 million children, adolescents, and adults. The Lancet [Internet]. 2024;403(10431):1027–50. Available from: 10.1016/S0140-6736(23)02750-2 10.1016/S0140-6736(23)02750-2 PMC7615769 38432237

[2] Angrisani L, Santonicola A, Iovino P, et al. IFSO worldwide survey 2020–2021: current trends for bariatric and metabolic procedures. Obes Surg. 2024;34(4):1075–85. 10.1007/s11695-024-07118-3. 38438667 10.1007/s11695-024-07118-3 PMC11026210

[3] Ong KL, Stafford LK, McLaughlin SA, et al. Global, regional, and national burden of diabetes from 1990 to 2021, with projections of prevalence to 2050: a systematic analysis for the Global Burden of Disease Study 2021. Lancet. 2023;402(10397):203–34. 10.1016/S0140-6736(23)01301-6. 10.1016/S0140-6736(23)01301-6 PMC10364581 37356446

[4] Mingrone G, Panunzi S, De Gaetano A, et al. Bariatric-metabolic surgery versus conventional medical treatment in obese patients with type 2 diabetes: 5 year follow-up of an open-label, single-centre, randomised controlled trial. Lancet. 2015;386(9997):964–73. 10.1016/S0140-6736(15)00075-6. 10.1016/S0140-6736(15)00075-6 26369473

[5] Svanevik M, Lorentzen J, Borgeraas H, et al. Patient-reported outcomes, weight loss, and remission of type 2 diabetes 3 years after gastric bypass and sleeve gastrectomy (Oseberg); a single-centre, randomised controlled trial. Lancet Diabetes Endocrinol. 2023;11(8):555–66. 10.1016/S2213-8587(23)00127-4. 10.1016/S2213-8587(23)00127-4 37414071

[6] Aminian A, Wilson R, Zajichek A, et al. Cardiovascular outcomes in patients with type 2 diabetes and obesity: comparison of gastric bypass, sleeve gastrectomy, and usual care. Diabetes Care. 2021;44(11):2552–63. 10.2337/dc20-3023. 10.2337/dc20-3023 34503954

[7] Closs C, Ackerman M, Masson W, et al. Effectiveness of Roux-en-Y gastric bypass vs sleeve gastrectomy on lipid levels in type 2 diabetes: a meta-analysis. J Gastrointest Surg. 2022;26(8):1575–84. 10.1007/s11605-022-05338-5. 10.1007/s11605-022-05338-5 35513608

[8] Ruiz-Tovar J, Vorwald P, Gonzalez-Ramirez G, et al. Impact of biliopancreatic limb length (70 cm vs 120 cm), with constant 150 cm alimentary limb, on long-term weight loss, remission of comorbidities and supplementation needs after Roux-en-Y gastric bypass: a prospective randomized clinical trial. Obes Surg. 2019;29(8):2367–72. 10.1007/s11695-019-03717-7. 10.1007/s11695-019-03717-7 31104282

[9] Steenackers N, Vanuytsel T, Augustijns P, et al. Adaptations in gastrointestinal physiology after sleeve gastrectomy and Roux-en-Y gastric bypass. Lancet Gastroenterol Hepatol. 2021;6(3):225–37. 10.1016/S2468-1253(20)30302-2 33581761

[10] Hindsø M, Svane MS, Hedbäck N, et al. The role of GLP-1 in postprandial glucose metabolism after bariatric surgery: a narrative review of human GLP-1 receptor antagonist studies. Surg Obes Relat Dis. 2021;17(7):1383–91. 10.1016/j.soard.2021.01.041 33771461

[11] Mika A, Kaska L, Proczko-Stepaniak M, et al. Evidence that the length of bile loop determines serum bile acid concentration and glycemic control after bariatric surgery. Obes Surg. 2018;28(11):3405–14. 10.1007/s11695-018-3314-9 29790128

[12] Shan CX, Qiu NC, Liu ME, et al. Effects of diet on bile acid metabolism and insulin resistance in type 2 diabetic rats after Roux-en-Y gastric bypass. Obes Surg. 2018;28(10):3044–53. 10.1007/s11695-018-3264-2. 10.1007/s11695-018-3264-2 29721762

[13] Kwon Y, Lee S, Kim D, et al. Biliopancreatic limb length as a potential key factor in superior glycemic outcomes after Roux-en-Y gastric bypass in patients with type 2 diabetes: a meta-analysis. Diabetes Care. 2022;45(12):3091–100. 10.2337/dc22-0835. 10.2337/dc22-0835 36455123

[14] Zerrweck C, Herrera A, Sepúlveda EM, et al. Long versus short biliopancreatic limb in Roux-en-Y gastric bypass: short-term results of a randomized clinical trial. Surg Obes Relat Dis. 2021;17(8):1425–30. 10.1016/j.soard.2021.03.030. 10.1016/j.soard.2021.03.030 33952426

[15] Aleassa EM, Papasavas P, Augustin T, et al. American Society for Metabolic and Bariatric Surgery literature review on the effect of Roux-en-Y gastric bypass limb lengths on outcomes. Surg Obes Relat Dis. 2023;19(7):755–62. 10.1016/j.soard.2023.04.298. 10.1016/j.soard.2023.04.298 37268517

[16] Bruinsma FFE, Nienhuijs SW, Liem RSL, et al. The impact of longer biliopancreatic limb length on weight loss and comorbidity improvement at 5 years after primary Roux-en-Y gastric bypass surgery: a population-based matched cohort study. Obes Surg. 2024;34(9):3236–45. 10.1007/s11695-024-07267-5. 10.1007/s11695-024-07267-5 PMC11349854 38981956

[17] Riddle MC, Cefalu WT, Evans PH, et al. Consensus report: definition and interpretation of remission in type 2 diabetes. Diabetes Care. 2021;44(10):2438–44. 10.2337/dci21-0034. 10.2337/dci21-0034 PMC8929179 34462270

[18] Koulas SG, Stefanou CK, Stefanou SK, et al. Gut microbiota in patients with morbid obesity before and after bariatric surgery: a ten-year review study (2009–2019). Obes Surg. 2020;31(1):317–26. 10.1007/s11695-020-05074-2 33130944

[19] Guo Y, Huang ZP, Liu CQ, et al. Modulation of the gut microbiome: a systematic review of the effect of bariatric surgery. Eur J Endocrinol. 2017;178(1):43–56. 10.1530/EJE-17-0403 28916564

[20] Nauck MA, Quast DR, Wefers J, et al. The evolving story of incretins (GIP and GLP-1) in metabolic and cardiovascular disease: a pathophysiological update. Diabetes Obes Metab. 2021;23(Suppl 3):5–29. 10.1111/dom.14496 34310013

[21] Alfaris N, Waldrop S, Johnson V, et al. GLP-1 single, dual, and triple receptor agonists for treating type 2 diabetes and obesity: a narrative review. EClinicalMedicine. 2024;75:102782. 10.1016/j.eclinm.2024.102782 PMC11402415 39281096

[22] Drucker DJ. GLP-1 physiology informs the pharmacotherapy of obesity. Mol Metab. 2021Oct;57:101351. 34626851 10.1016/j.molmet.2021.101351 PMC8859548

[23] Camacho-Ramírez A, Prada-Oliveira JA, Ribelles-García A, et al. The leading role of peptide tyrosine tyrosine in glycemic control after Roux-en-Y gastric bypass in rats. Obes Surg U.S. 2020;30(2):697–706. 10.1007/s11695-019-04239-y. 10.1007/s11695-019-04239-y 31701411

[24] Zhang C, Zhang J, Zhou Z. Changes in fasting bile acid profiles after Roux-en-Y gastric bypass and sleeve gastrectomy. Medicine (Baltimore). 2021;100(3):e23939. 33545968 10.1097/MD.0000000000023939 PMC7837931

[25] Patrício BG, Morais T, Guimarães M, et al. Gut hormone release after gastric bypass depends on the length of the biliopancreatic limb. Int J Obes (Lond). 2018;43(5):1009–18. 10.1038/s41366-018-0117-y 29795464

[26] Pal A, Rhoads DB, Tavakkoli A. Customization of biliopancreatic limb length to modulate and sustain antidiabetic effect of gastric bypass surgery. Am J Physiol Gastrointest Liver Physiol [Internet]. 2017;314(2):G287–99. Available from: 10.1152/ajpgi.00276.2017 10.1152/ajpgi.00276.2017 PMC5866424 29097359

[27] Nora M, Morais T, Almeida R, et al. Should Roux-en-Y gastric bypass biliopancreatic limb length be tailored to achieve improved diabetes outcomes? Medicine (Baltimore). 2017;96(48):e8859. 10.1097/MD.0000000000008859 PMC5728768 29310367

[28] Aung L, Lee WJ, Chen SC, et al. Bariatric surgery for patients with early-onset vs late-onset type 2 diabetes. JAMA Surg. 2016;151(9):798–805. 10.1001/jamasurg.2016.1130. 10.1001/jamasurg.2016.1130 27248572

[29] Fultang J, Chinaka U, Rankin J, et al. Preoperative bariatric surgery predictors of type 2 diabetes remission. J Obes Metab Syndr. 2021;30(2):104–14. 10.7570/jomes20084 PMC8277586 33436532

[30] Haddad A. Comment on: Type 2 diabetes remission after Roux-en-Y gastric bypass: a multicentered experience with long-term follow-up. Surg Obes Relat Dis. 2023Nov;20(6):e3–4. 37989623 10.1016/j.soard.2023.10.011

[31] Panunzi S, Carlsson L, De Gaetano A, et al. Determinants of diabetes remission and glycemic control after bariatric surgery. Diabetes Care. 2015;39(1):166–74. 10.2337/dc15-0575 26628418

[32] Eisenberg D, Shikora SA, Aarts E, et al. 2022 American Society of Metabolic and Bariatric Surgery (ASMBS) and International Federation for the Surgery of Obesity and Metabolic Disorders (IFSO) indications for metabolic and bariatric surgery. Obes Surg. 2023;33(1):3–14. 10.1007/s11695-022-06332-1 PMC9834364 36336720

[33] Silveira FC, Docherty NG, Sallet PC, et al. Early post-operative weight change after Roux-en-Y gastric bypass predicts weight loss at 12-month follow-up. Obes Surg. 2020;30(12):5020–5. 10.1007/s11695-020-04942-1. 10.1007/s11695-020-04942-1 32857300

[34] Krebs NF. Overview of zinc absorption and excretion in the human gastrointestinal tract. J Nutr. 2000May;130(5):1374S-1377S. 10801946 10.1093/jn/130.5.1374S

[35] Thereaux J, Lesuffleur T, Czernichow S, et al. Long-term adverse events after sleeve gastrectomy or gastric bypass: a 7-year nationwide, observational, population-based, cohort study. Lancet Diabetes Endocrinol. 2019;7(10):786–95. 10.1016/S2213-8587(19)30191-3 31383618

[36] Nergaard BJ, Leifsson BG, Hedenbro J, et al. Gastric bypass with long alimentary limb or long pancreato-biliary limb–long-term results on weight loss, resolution of co-morbidities and metabolic parameters. Obes Surg. 2014;24(10):1595–602. 10.1007/s11695-014-1245-7. 10.1007/s11695-014-1245-7 PMC4153949 24744188

[37] Rizvi SHA, Jamil DK, Rohail S, et al. Efficacy and safety of long vs short biliopancreatic limb in Roux-en-Y gastric bypass surgery: a systematic review and meta-analysis. Curr Probl Surg. 2024;61(10):101562. 10.1016/j.cpsurg.2024.101562. 10.1016/j.cpsurg.2024.101562 39266128

[38] Kraljević M, Schneider R, Wölnerhanssen B, et al. Different limb lengths in gastric bypass surgery: study protocol for a Swiss multicenter randomized controlled trial (SLIM). Trials. 2021;22(1):352. 10.1186/s13063-021-05313-6 PMC8136210 34011386

